# Efficacy evaluation of standardized *Rheum rhaponticum* root extract (ERr 731^®^) on symptoms of menopause: A systematic review and meta-analysis study

**DOI:** 10.7555/JBR.37.20230219

**Published:** 2024-04-18

**Authors:** Vishal P. Dubey, Varun P. Sureja, Dharmeshkumar B. Kheni

**Affiliations:** Department of Scientific and Medical Affairs, Sundyota Numandis Probioceuticals Pvt. Ltd., Ahmedabad, Gujarat 380015, India

**Keywords:** menopause, *Rheum rhaponticum*, hot flashes, systematic review

## Abstract

Menopause is characterized by various physical, mental and emotional symptoms. ERr 731^®^ is a standardized extract from *Rheum rhaponticum* root and has been clinically studied for its role in reducing menopausal symptoms. The current systematic review and meta-analysis aimed to evaluate the efficacy of ERr 731^®^ supplementation in alleviating the severity of menopausal symptoms. In this review, we searched across three online databases up to March 2023, evaluated the quality of the included studies by the Physiotherapy Evidence Database scale, and assessed the risk of bias by the Cochrane Risk of Bias tool. We then performed a meta-analysis using RevMan software to estimate the pooled mean difference (MD). The study protocol was registered in the Prospective Register of Systematic Reviews (CRD42023416808). After screening and evaluation, we included four high-quality studies (a total of 390 participants; the ERr 731^®^ group: 193 participants; the control group: 197 participants) in the meta-analysis. The results showed that ERr 731^®^ supplementation significantly reduced the Menopause Rating Scale score (MD: –15.12; *P* < 0.001), compared with control therapy. Sensitivity analysis revealed no effect of individual studies on the overall pooled estimate or overall observed heterogeneity. The current review provides evidence that ERr 731^®^ supplementation is effective in reducing menopause symptoms. Potential bias and high heterogeneity in the results warrant further clinical studies.

## Introduction

Menopause is the natural cessation of a normal menstrual cycle for 12 consecutive months, indicating the end of the reproductive phase in females. The menopausal transition is defined as the period of physiological changes observed in women as they approach reproductive aging^[1–2]^. During this transition period, significant hormonal fluctuations are observed, primarily in the levels of estrogen and progesterone, which sharply decline at menopause and result in various physical, mental and emotional symptoms collectively termed climacteric symptoms^[1–3]^. Menopause generally occurs between the ages of 45 and 55, and more than 70% of the subjects have impaired quality of life (QoL)^[4]^. All these lines of evidence suggest that a large population of women live with menopausal symptoms from an early age and will have to spend almost 30 years of their lives dealing with all the menopausal complications.

The hormonal changes during menopause result in various physical, psychological, and emotional changes that manifest as different symptoms, including hot flashes, night sweats, mood swings and irritability, vaginal dryness and discomfort during sexual intercourse, sleep disturbances, weight gain, slowed metabolism, joint pain, muscle pain and stiffness, memory and concentration problems, urinary incontinence, frequent urination, and loss of libido and sexual function^[5]^. Additionally, various studies have demonstrated that metabolic syndrome significantly increases the severity of menopause symptoms^[1]^. Other factors, including an increased level of triglycerides and testosterone but a reduced level of progesterone, are shown to have a positive correlation with the severity of menopause symptoms^[2]^.

Current treatment options for reducing the severity of climacteric symptoms include exogenous hormonal therapies (estrogen therapy either alone or in combination with progesterone) and phytoestrogens (like isoflavones and black cohosh extract)^[6–7]^. However, the use of exogenous hormonal therapies is associated with an elevated risk of cancer, thromboembolism, and cardiovascular side effects^[8]^. Similarly, phytoestrogen therapies lack long-term safety and efficacy data and are also associated with an elevated risk of potential side effects^[7]^.

**Figure 1:**
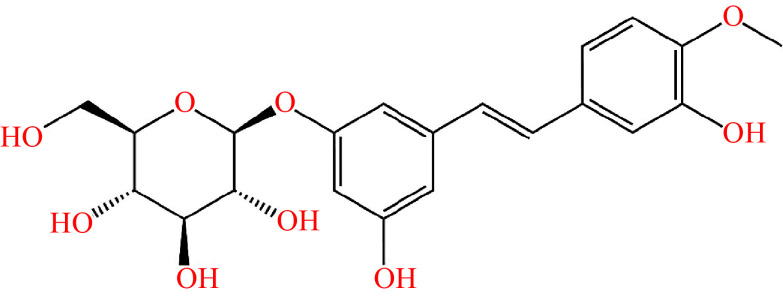
Structure of rhaponticin.

*Rheum rhaponticum*, also known as *Rhapontic rhubarb*, has been traditionally used for the treatment of menopausal symptoms^[9]^. The root of *R. rhaponticum* contains various phytoconstituents, mainly rhaponticin (chemical structure depicted in ***Fig. 1***), which is believed to reduce the severity of climacteric symptoms^[9]^. ERr 731^®^ is a standardized *R. rhaponticum* root extract that has been clinically evaluated for its potential use in the management of menopausal symptoms^[5]^. However, the literature lacks a dedicated systematic review and meta-analysis to evaluate the effectiveness of ERr 731^®^ supplementation in reducing the severity of menopausal symptoms. Hence, we conducted the current systematic review and meta-analysis of randomized controlled studies to examine the efficacy of standardized *R. rhaponticum* root extract (ERr 731^®^) in reducing the severity of menopausal symptoms.

## Materials and methods

### The study conduct and protocol registration

The current systematic review and meta-analysis was conducted and reported following the Preferred Reporting Items for Systematic Reviews and Meta-Analyses (PRISMA) guidelines^[10]^, the Cochrane Handbook for Systematic Reviews of Interventions, and the Cochrane Statistical Methods guidelines^[11]^. The review methods were previously established before the initiation of the review. The study protocol has been registered and can be accessed at the Prospective Register of Systematic Reviews (PROSPERO) with the registration number CRD42023416808.

### Review question

The review question was framed based on PICOS (population, interventions, comparators, outcomes, and study design) criteria (***Table 1***) as follows: does standardized *R. rhaponticum* root extract supplementation effectively reduce the severity of climacteric symptoms in perimenopause and/or menopause subjects?

**Table 1:** PICOS criteria for study determination

Parameters	Description
Population	Peri-menopausal and/or post-menopausal female subjects suffering from climacteric symptoms
Intervention	Nutraceutical/dietary supplement/herbal supplement/medicinal food containing *R. rhaponticum* root extract (either alone or in combination)
Comparison	Either placebo or any supplement without *R. rhaponticum* root extract (either alone or in combination)
Outcomes	Efficacy of supplement evaluated using the Menopause Rating Scale score
Study design	Randomized, controlled clinical study with parallel or cross-over design

### Information sources and search strategy

All scientific publications indexed in the English-language databases of Google Scholar, PubMed/MEDLINE, and ScienceDirect before March 2023, were systematically evaluated to identify studies that might be eligible and could be included in the current study. The terms used alone or in combination for the literature search were: ("rheum rhaponticum" OR "ERr 731") AND ("menopause" OR "menopaus* symptom") AND ("female" OR "women") AND ("menopause rating scale" OR "MRS") AND ("randomized trial" OR "randomized study" OR "controlled trial" OR "controlled study"). In addition, the reference lists of relevant publications were manually examined to identify any grey literature that could be included in the current study.

### Eligibility criteria

All randomized controlled trials evaluating the efficacy of ERr 731^®^ in the subjects with symptoms of menopause, and articles available in the English language and evaluating the efficacy of interventions using the Menopause Rating Scale (MRS) score were considered eligible. Non-randomized, single-arm studies or studies with a different design from that described in the inclusion criteria, studies evaluating the efficacy of *Rheum* species other than the standardized *R. rhaponticum* root extract, studies evaluating the efficacy of interventions with the evaluating parameters different from the MRS score, and studies available in languages other than English were excluded from the review.

### Study selection and quality assessment

All articles retrieved by the literature search were initially evaluated based on their titles and abstracts. Unrelated studies were excluded. All studies that met the eligibility criteria or were deemed eligible for inclusion in the study were further evaluated using the full text of the articles.

The internal and external validity and the statistical sufficiency of the included studies were assessed by the Physiotherapy Evidence Database (PEDro) scale^[12–13]^. The PEDro scale categorizes studies into three categories based on an 11-point scoring system: high quality (≥ 8 points), moderate quality (4 to 7 points), and low quality (≤ 3 points)^[12]^.

After eligibility confirmation, the study characteristics were collected, including lead author, publication year, indication, sample size, age, interventions provided, and duration of the study. Data regarding the effect of interventions on the MRS-total score were also collected from each study.

### Data collection

The following data were extracted from each eligible study using a pre-designed datasheet: authors' names, year of publication, type of active intervention provided, type of control intervention provided, number of participants in each group, age of participants, and duration of the study. The outcome measure for the review was the change in the MRS score from the baseline to the end of the study with the provided interventions. The outcome measure was collected on a separate pre-designed sheet from each study.

### Risk of bias assessment

The methodological quality of all included studies was assessed using the Cochrane Risk of Bias (RoB) tool^[14]^. This tool assesses overall bias based on five different domains: randomization process, deviations from intended interventions, missing outcome data, measurement of the outcome, and selection of reported result. The tool algorithm generated an outcome of low, some concern, or high risk of bias, along with the overall judgment. Independent risk of bias analysis was conducted by the authors, and any disagreements were resolved by joint consensus.

### Statistical analysis

We used RevMan software (Desktop v5.4.1) provided by the Cochrane collaboration network for conducting statistical analysis to derive the forest plot showing the results of individual studies and the pooled analysis estimate. The mean difference and standard deviation (change from baseline) (*SD*_change_) from the included studies were used for continuous evaluation, with a 95% confidence interval (CI). The *SD*_change_ for individual parameters was adopted from respective studies, and if the *SD*_change_ was not provided, it was estimated using the following formula from the Cochrane Handbook for Systematic Reviews of Interventions^[11]^.

$
S  D_{{\mathrm{change}} }=\sqrt{\left(S  D_{\mathrm{B}}^2+S  D_{\mathrm{F}}^2\right)-\left(2 \times r \times S  D_{\mathrm{B}} \times S  D_{\mathrm{F}}\right)}$

Where "SD_B_" and "SD_F_" denote the standard deviation at baseline and final visit, respectively, while "r" denotes the correlation coefficient, either obtained from other studies or considered to be 0.7 to provide a conservative estimate, as undertaken from previous studies^[15]^. The heterogeneity of the included studies was assessed by the Higgins *I*^2^ index, with *I*^2^ > 50% indicating significant heterogeneity. The fixed-effects model was used in case of a low heterogeneity, and the random-effects model was used in case of a high heterogeneity. Meta-essential (v1.5) was used for evaluating the publication bias by the Egger regression test and Begg-Mazumdar test. The trimming and filling method was used in instances of significant publication bias to identify any missing studies and assess their effects on the overall effect size (Cohen's *d* value). The funnel plot was plotted to visualize the publication bias and evaluate the effect of any missing studies on the pooled effect size. Sensitivity analysis was conducted by the leave-one-study-out method to determine the effect of individual studies on both the estimated pooled effect and the overall observed heterogeneity, using OpenMeta-Analyst software. A *P*-value of < 0.05 was considered statistically significant.

## Results

### Study selection and study characteristics

**Figure 2:**
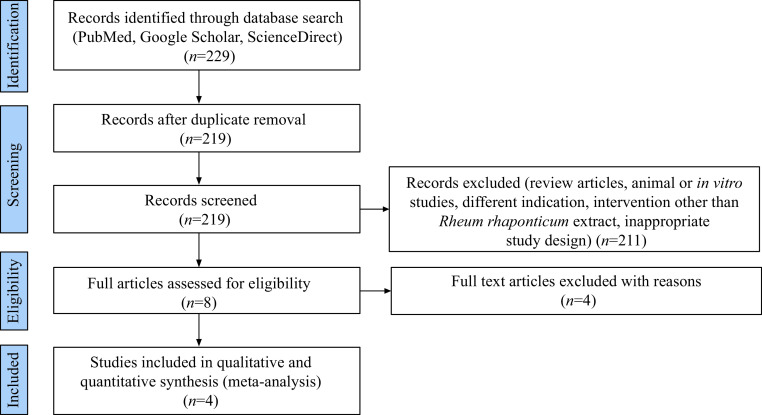
Flowchart of the study selection and inclusion process following the Preferred Reporting Items for Systematic Reviews and Meta-Analyses (PRISMA) guidelines.

We identified 229 records up to March 2023. After removing duplicate articles, 219 articles were selected for initial screening. The title and abstract screening removed 211 articles, and eight articles were assessed using full-text screening for final eligibility. Among these, four articles were included in the final qualitative and quantitative analysis^[16–19]^. The detailed study selection process is presented in ***Fig. 2***. The study characteristics are presented in ***Table 2***. Data from 390 participants (193 participants in the active therapy group and 197 participants in the control therapy group) were used for meta-analysis.

**Table 2:** Characteristics of the included studies

Studies	ERr 731^®^ therapy (*n*; age [years, mean±SD])	Placebo therapy (*n*; age [years, mean±SD])	Study duration (weeks)
Heger M *et al.*, 2006^[16]^	54; 49.3±3.0	55; 48.6±3.1	12
Kaszkin-Bettag M *et al.*, 2009^[17]^	56; 49.4±3.6	56; 49.6±3.0	12
Hasper I *et al.*, 2009^[18]^	39; 49.5±2.8	41; 49.0±3.2	12
Thiemann E *et al.*, 2017^[19]^	44^a^	45^a^	12
^a^Data regarding the age of participants is not available.Abbreviation: SD, standard deviation.			

**Figure 3:**
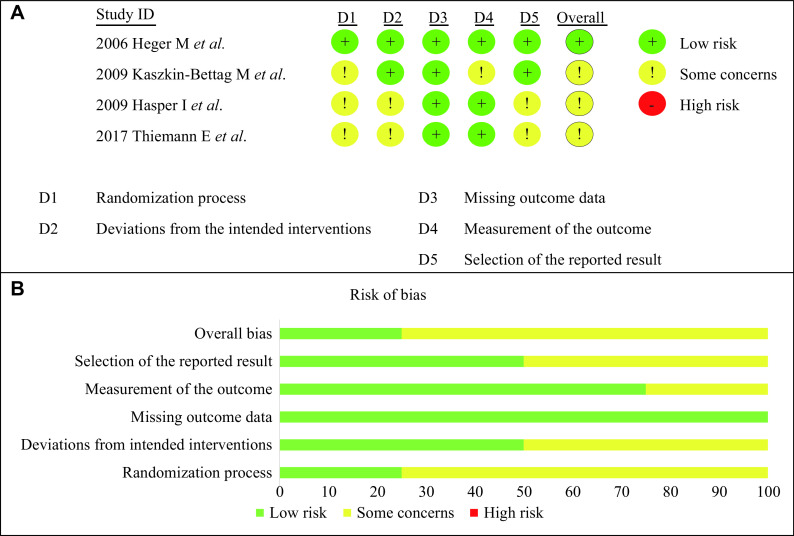
Individual and overall risk of bias assessment.

The results of the overall risk of bias assessment are presented in ***Fig. 3***, and the results of the study quality evaluated by the PEDro scale are presented in ***Table 3*** (detailed assessments of the quality evaluation provided in ***Supplementary Table 1*** [available online]). All the included studies were of good quality.

**Table 3:** Quality assessment of the included studies (PEDro scale)

Parameters	Studies			
	Heger M *et al.*^[16]^	Kaszkin-Bettag M *et al.*^[17]^	Hasper I *et al.*^[18]^	Thiemann E *et al.*^[19]^
Eligibility criteria	1	1	1	0
Random allocation	1	1	1	1
Concealed allocation	1	0	0	0
Groups similar	1	1	1	1
Subject blinding	1	1	1	1
Therapist blinding	1	1	1	1
Assessor blinding	1	1	1	1
Less than 15% of dropouts	0	1	0	0
Intention-to-treat	1	1	1	1
Between-group statistical evaluation	1	1	1	1
Point measures	1	1	1	1
Overall score	10	10	9	8
The scale categorizes the included studies into three categories: high quality (≥ 8 points), moderate quality (4–7 points), and low quality (≤ 3 points). Abbreviation: PEDro, Physiotherapy Evidence Database.				

Overall, all the included studies were randomized placebo-controlled studies, and the participants were divided into groups with similar baseline characteristics. All included studies ensured complete blinding of the subjects, therapists, and assessors who were included in the study. All the included studies adopted the intention-to-treat analysis method for the final analysis, and all the included studies provided the results of between-group statistical comparisons and the point as well as variable data for the main outcome parameter (the MRS score).

### Result of meta-analysis

**Figure 4:**
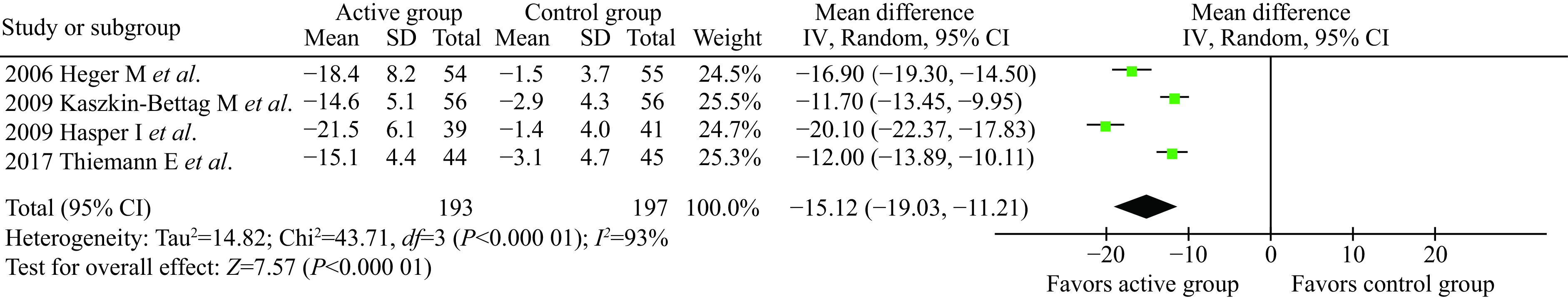
Results of meta-analysis.

The effect of ERr 731^®^ supplementation on reducing symptoms of menopause is depicted in ***Fig. 4***. ERr 731^®^ significantly reduced the MRS score (mean difference: –15.12; 95% CI: –19.03 to –11.21; *P* < 0.001), compared with the control group. A high level of heterogeneity was observed among the included studies (*I^2^* = 93%), thus a random-effects model was used to present the pooled analysis.

### Sensitivity analysis

The extent of heterogeneity was evaluated by conducting a sensitivity analysis using the leave-one-study-out analysis. The results of the analysis are presented in ***Table 4***. The removal of individual studies had no significant effect on the overall observed heterogeneity and the estimated pooled estimate value.

**Table 4:** Effects of individual study on overall mean difference pooled analysis and overall heterogeneity

Studies excluded	*I* ^*2*^	Mean difference estimate	95% CI	*P*
Heger M *et al.*^[16]^	95%	−14.55	−19.52 to −9.59	<0.001
Kaszkin-Bettag M *et al.*^[17]^	93%	−16.30	−21.18 to −11.42	<0.001
Hasper I *et al.*^[18]^	85%	−13.43	−16.41 to −10.46	<0.001
Thiemann E *et al.*^[19]^	94%	−16.19	−21.33 to −11.05	<0.001
Overall	93%	−15.12	−19.03 to −11.21	<0.001
Sensitivity analysis was conducted by leave-one-study-out analysis. The effect of individual study on observed heterogeneity was assessed by Higgin's index value (*I*^*2*^), while the effect on the pooled estimate was evaluated by mean difference estimate value. *P* < 0.05 is considered statistically significant. Abbreviation: CI, confidence interval.				

### Publication bias assessment

**Figure 5:**
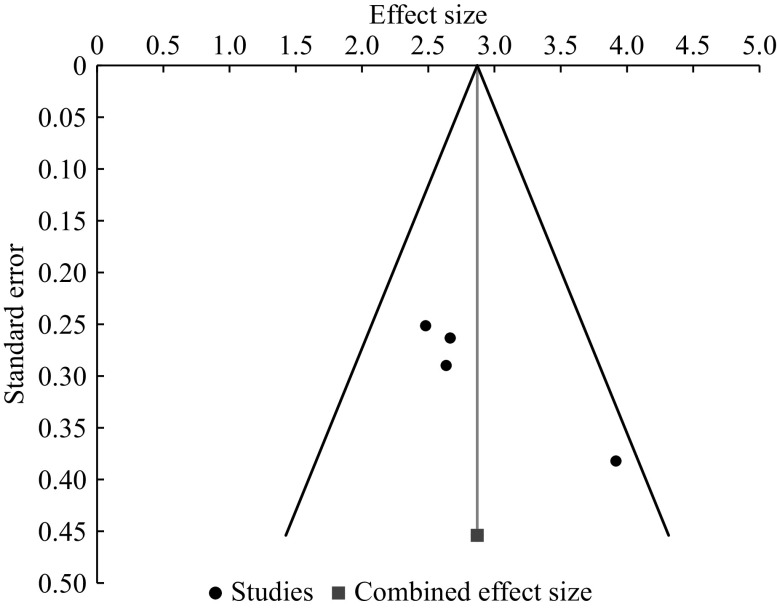
A funnel plot for evaluating publication bias.

The assessment of publication bias using the random effect model showed a significant publication bias based on the Egger regression test (*P* = 0.022). However, no significance was observed when using the Begg-Mazumdar test. The Cohen's *d* value and random effect model were also non-significant (*P* = 0.174). The trimming and filling analysis for the missing study identification revealed no missing studies. The results of the publication bias are depicted through a funnel plot in ***Fig. 5***.

## Discussion

### Findings and interpretations

The current systematic review and meta-analysis study indicates that ERr 731^®^ supplementation is effective in relieving menopausal symptoms.

Estrogen has a variety of functions in the body exerted through estrogen receptors (ERs), namely ER-α and ER-β^[20]^. These receptors are widely distributed in the body and have various functions^[21]^. Hence, in menopause, the decreased level of estrogen causes a variety of symptoms affecting different aspects of the body. While the supplementation of exogenous estrogen and phytoestrogens has been implicated in reducing the severity of menopause symptoms, the overaction of ER-α leads to an increased risk of cancer, whereas the activation of ER-β suppresses the proliferation of these tumor cells^[22–23]^.

The root extract of *R. rhaponticum* has been used in Germany for decades to reduce climacteric symptoms of peri- and post-menopausal women^[24]^. ERr 731^®^ is a standardized *R. rhaponticum* root extract that contains rhaponticin as the active ingredient^[25]^. ERr 731^®^ specifically binds to ER-β without activating ER-α, thereby causing a decrease in menopausal symptom severity without any potential side effects^[24]^. The mechanism of action of ERr 731^®^ was evaluated by two independent *in vitro* studies, supporting the ER-β specific activity of ERr 731^®[24,26]^. Pre-clinical studies have evaluated the safety of ERr 731^®^ in an ovariectomized animal model, in which ERr 731^®^ supplementation has no uterine and endometrial proliferative action, while concomitant estrogen supplementation increases uterine and endometrial proliferation^[27–29]^. Additionally, a long-term safety study in dogs showed that the no-observed-adverse-effect-level for ERr 731^®^ reached up to 1000 mg/kg body weight per day^[30]^.

### Comparison with other studies

The results of the current study are novel, because it is the first systematic review and meta-analysis study to evaluate the efficacy of *R. rhaponticum* extract in peri- and post-menopausal women. Various clinical studies have evaluated the efficacy of *R. rhaponticum* root extract in managing climacteric symptoms. In a study of perimenopausal women with climacteric symptoms, supplementation of *R. rhaponticum* root extract for 12 weeks resulted in a significant reduction in the severity of climacteric symptoms and improvement in overall QoL, compared with placebo supplementation, while none of the subjects had any adverse effects or uterine/endometrial-related abnormalities^[16]^. The post-hoc evaluation of the study was published separately, in which the *R. rhaponticum* root extract supplementation was found to significantly reduce anxiety and depression in perimenopausal subjects, compared with the placebo group^[25]^. In a similar study, supplementation of *R. rhaponticum* root extract was associated with a significant reduction in the MRS score and significantly improved treatment outcomes in perimenopausal women^[17]^. In a six-month open observational study, *R. rhaponticum* root extract supplementation in menopausal women resulted in a significant reduction in the MRS score and significantly improved QoL^[31]^. Another clinical study was conducted for a two-year duration, in which *R. rhaponticum* root extract supplementation was associated with a significant reduction in all major climacteric symptoms without any side effects^[18]^. These findings indicate that ERr 731^®^ is a safe therapy for treating menopause symptoms that may be effectively used for a longer period without any risk of adverse events. The safety of *R. rhaponticum* root extract has also been evaluated in a post-marketing safety surveillance study^[32]^, and the study found that out of the 153.12 million doses consumed in Germany between 1993 and 2014, in North America between 2009 and 2014, and in South Africa between 2011 and 2014 combined, the number of adverse events reported was very low, concluding that the consumption of *R. rhaponticum* root extract was completely safe^[32]^.

### Strengths and limitations of the study

The strengths of the current study include the following: firstly, being the only study that evaluated the effectiveness of *R. rhaponticum* extract in reducing climacteric symptoms with a systematic review and meta-analysis design; secondly, the results of the current study are in line with those of previously published clinical studies; transparency is also an important strength of the current study, which was maintained by following the guidelines provided by PRISMA and registering the study protocol in PROSPERO. The limitations of the current study include, firstly, the high heterogeneity observed in the pooled analysis. We conducted a leave-one-study-out analysis to assess the effect of individual studies on overall heterogeneity. However, the observed heterogeneity might be attributed to other factors that need to be addressed by conducting more rigorous studies. Secondly, the low-to-moderate risk of bias observed among the included studies needs to be addressed by conducting more well-designed, transparent clinical studies. The significant reporting bias observed in the study by using the Egger test but not in the Begg-Mazumdar test needs further attention. Lastly, the study evaluated the efficacy of *R. rhaponticum* extract on the overall MRS score but could not evaluate the effect of *R. rhaponticum* supplement on individual symptoms evaluated by using MRS because of the unavailability of data. Despite the limitations of the study, the results of the current study are promising and warrant further clinical studies to evaluate the efficacy of ERr 731^®^ in peri- and post-menopausal women.

In conclusion, the current systematic review and meta-analysis study suggests that ERr 731^®^ supplementation is effective in reducing menopause symptoms. However, there are concerns regarding the risk of bias and high heterogeneity among the included studies. These limitations need to be addressed in future studies, and more high-quality clinical studies are required to validate the results of the current study.

## SUPPLEMENTARY DATA

Supplementary data to this article can be found online.
