# Supplementary material for: Efficacy evaluation of standardized Rheum rhaponticum root extract (ERr 731®) on symptoms of menopause: A systematic review and meta-analysis study
Source: J Biomed Res. 2024 Apr 18;38(3):278–86. doi: 10.7555/JBR.37.20230219 (PMC11144934; doi:10.7555/JBR.37.20230219)
Supplement: Supplementary file 1 — Supplementary data to this article can be found online. [file jbr-38-3-278-S1.pdf]

Available online at www.jbr-pub.org.cn

Open Access at PubMed Central

JBR

Journal of Biomedical Research, 2024 0(000): 1–3

*Supplementary Data*

# Efficacy evaluation of standardized *Rheum rhaponticum* root extract (ERr 731®) on symptoms of menopause: A systematic review and meta-analysis study

Vishal P. Dubey✉, Varun P. Sureja, Dharmeshkumar B. Kheni

*Department of Scientific and Medical Affairs, Sundyota Numandis Probioceuticals Pvt. Ltd., Ahmedabad, Gujarat 380015, India.*

***Supplementary Table 1*** **Judgement and support for the judgment of each included study in the PEDro scale**

| Entries | Judgment (Yes/No) | Support for judgment | |
|---|---|---|---|
| | | Quote | Comment of author |
| Heger M *et al.*, 2006 | | | |
| Eligibility criteria specified | Yes | "Inclusion criteria were (1) climacteric complaints…" | Eligibility criteria were specified. |
| Random allocation | Yes | "Women were randomized to either …" | Subjects were randomly allocated. |
| Concealed allocation | Yes | "Both participants and investigators and the data monitoring committee were blinded with regard to the individual treatment allocation." | Concealment was maintained. |
| Groups similar at baseline | Yes | "None of these characteristics, including serum hormone levels, differed significantly between the treatment groups." | Groups were similar at baseline. |
| Subject blinding | Yes | "Both participants and investigators and the data monitoring committee were blinded with regard to the individual treatment allocation." | Subjects were blinded and the procedures were informed. |
| Therapist blinding | Yes | "Both participants and investigators and the data monitoring committee were blinded with regard to the individual treatment allocation." | Therapists were blinded and the procedures were informed. |
| Assessor blinding | Yes | "Both participants and investigators and the data monitoring committee were blinded with regard to the individual treatment allocation." | Subjective scales were used, hence assessor was blinded, and procedures were informed. |
| Less than 15% dropouts | No | As shown in ***Table 2*** of study article | More than 15% dropouts observed in any one of the interventional groups. |
| Intention-to-treat analysis | Yes | "109 women, 54 in the ERr 731 group and 55 in the placebo group, were included in the intention-to-treat population." | Intention-to-treat analysis method was used. |

✉Corresponding author: Vishal P. Dubey, Department of Scientific and Medical Affairs, Sundyota Numandis Probioceuticals Pvt. Ltd., Prahladnagar, Ahmedabad, Gujarat 380015, India. E-mails: vishal.d@sundyotanumandis.com and rx.vishaldubey@gmail.com.

Received: 15 September 2023; Revised: 16 January 2024; Accepted: 22 January 2024; Published online: 18 April 2024

CLC number: R711, Document code: A

The authors reported no conflict of interests.

This is an open access article under the Creative Commons Attribution (CC BY 4.0) license, which permits others to distribute, remix, adapt and build upon this work, for commercial use, provided the original work is properly cited.

© 2024 by Journal of Biomedical Research.

https://doi.org/10.7555/JBR.37.20230219

***Supplementary Table 1*** **Judgement and support for the judgment for each included study in the PEDro scale** (continued)

| Entries | Judgment (Yes/no) | Support for judgment | |
|---|---|---|---|
| | | Quote | Comment of author |
| Between-group statistical comparison | Yes | Described in detail in study article | Between-group statistical analysis was performed, and results are reported. |
| Point measures and variability | Yes | Described in detail in study article | Data of point measure and measure of variability for at least one key outcome were reported. |
| Kaszkin-Bettag M *et al.*, 2009 | | | |
| Eligibility criteria specified | Yes | "Inclusion and exclusion criteria" | Eligibility criteria were specified. |
| Random allocation | Yes | "All enrolled trial subjects were randomized to treatment …" | Subjects were randomly allocated. |
| Concealed allocation | No | Not applicable | No details regarding randomization concealment provided. |
| Groups similar at baseline | Yes | "None of the baseline characteristics differed markedly between the treatment groups …" | Groups were similar at baseline. |
| Subject blinding | Yes | "This was a 12-week, multicenter, prospective, randomized, double-blind, parallel-group, placebo-controlled, phase Ⅲ clinical trial …" | Subjects were blinded and the procedures were informed. |
| Therapist blinding | Yes | "This was a 12-week, multicenter, prospective, randomized, double-blind, parallel-group, placebo-controlled, phase Ⅲ clinical trial …" | Therapists were blinded and the procedures were informed. |
| Assessor blinding | Yes | "This was a 12-week, multicenter, prospective, randomized, double-blind, parallel-group, placebo-controlled, phase Ⅲ clinical trial …" | Subjective scales were used, hence assessor was blinded, and procedures were informed. |
| Less than 15% dropouts | Yes | As shown in ***Fig. 1*** of study article | Data from more than 85% of the subjects initially allocated to groups were obtained for at least one key outcome. |
| Intention-to-treat analysis | Yes | "All women in the ERr 731 and the placebo group were included in the intention-to-treat analysis …" | Intention-to-treat analysis method was used. |
| Between-group statistical comparison | Yes | Described in detail in study article | Between-group statistical analysis was performed, and results are reported. |
| Point measures and variability | Yes | Described in detail in study article | Data of point measure and measure of variability for at least one key outcome were reported. |
| Hasper I *et al.*, 2009 | | | |
| Eligibility criteria specified | Yes | "Inclusion criteria for the RCT were …" | Eligibility criteria were specified. |
| Random allocation | Yes | "A total of 110 women enrolled in the trial were randomized to one of the two treatment groups …" | Subjects were randomly allocated. |
| Concealed allocation | No | Not applicable | No details regarding randomization concealment provided. |
| Groups similar at baseline | Yes | "At baseline (day 0 of the RCT), no remarkable differences in age, height, weight, and BMI, …" | Groups were similar at baseline. |
| Subject blinding | Yes | "The initial trial was a 12-week multicenter, prospective, randomized, double-blind, placebo-controlled, phase Ⅲ clinical trial …" | Subjects were blinded and the procedures were informed. |
| Therapist blinding | Yes | "The initial trial was a 12-week multicenter, prospective, randomized, double-blind, placebo-controlled, phase Ⅲ clinical trial …" | Therapists were blinded and the procedures were informed. |
| Assessor blinding | Yes | "The initial trial was a 12-week multicenter, prospective, randomized, double-blind, placebo-controlled, phase III clinical trial …" | Subjective scales were used, hence assessor was blinded, and procedures were informed. |

***Supplementary Table 1*** **Judgement and support for the judgment for each included study in the PEDro scale** (continued)

| Entries | Judgment (Yes/No) | Support for judgment | |
|---|---|---|---|
| | | Quote | Comment of author |
| Less than 15% dropouts | No | Not applicable | No accurate details regarding dropouts are provided. |
| Intention-to-treat analysis | Yes | "The intention-to-treat data set comprised all enrolled women who appeared for at least the first observational contact." | Intention-to-treat analysis method was used. |
| Between-group statistical comparison | Yes | Described in detail in study article | Between-group statistical analysis was performed, and results are reported. |
| Point measures and variability | Yes | Described in detail in study article | Data of point measure and measure of variability for at least one key outcome were reported. |
| Thiemann E *et al.*, 2017 | | | |
| Eligibility criteria specified | No | Not applicable | Eligibility criteria not specified. |
| Random allocation | Yes | "12-week double-blind, randomized placebo-controlled, prospective phase Ⅲ clinical trial …" | Subjects were randomly allocated. |
| Concealed allocation | No | Not applicable | No details regarding randomization concealment provided. |
| Groups similar at baseline | Yes | "At screening, no remarkable clinical differences …" | Groups were similar at baseline. |
| Subject blinding | Yes | "12-week double-blind, randomized placebo-controlled, prospective phase Ⅲ clinical trial …" | Subjects were blinded and the procedures were informed. |
| Therapist blinding | Yes | "12-week double-blind, randomized placebo-controlled, prospective phase Ⅲ clinical trial …" | Therapists were blinded and the procedures were informed. |
| Assessor blinding | Yes | "12-week double-blind, randomized placebo-controlled, prospective phase Ⅲ clinical trial …" | Subjective scales were used, hence assessor was blinded, and procedures were informed. |
| Less than 15% dropouts | No | Not applicable | No accurate details regarding dropouts are provided. |
| Intention-to-treat analysis | Yes | As shown in ***Table 1*** of study article | Intention-to-treat analysis method was used. |
| Between-group statistical comparison | Yes | Described in detail in study article | Between-group statistical analysis was performed, and results are reported. |
| Point measures and variability | Yes | Described in detail in study article | Data of point measure and measure of variability for at least one key outcome were reported. |
